# Giving meaning to tweets in emergency situations: a semantic approach for filtering and visualizing social data

**DOI:** 10.1186/s40064-016-3384-x

**Published:** 2016-10-13

**Authors:** Teresa Onorati, Paloma Díaz

**Affiliations:** Computer Science Department, Universidad Carlos III de Madrid, Avda de la Universidad 30, 28911 Leganes, Spain

**Keywords:** Information visualization, Information categorization, Emergency management, Semantic modeling, Ontologies

## Abstract

In this paper, we propose a semantic approach for monitoring information published on social networks about a specific event. In the era of Big Data, when an emergency occurs information posted on social networks becomes more and more helpful for emergency operators. As direct witnesses of the situation, people share photos, videos or text messages about events that call their attention. In the emergency operation center, these data can be collected and integrated within the management process to improve the overall understanding of the situation and in particular of the citizen reactions. To support the tracking and analyzing of social network activities, there are already monitoring tools that combine visualization techniques with geographical maps. However, tweets are written from the perspective of citizens and the information they provide might be inaccurate, irrelevant or false. Our approach tries to deal with data relevance proposing an innovative ontology-based method for filtering tweets and extracting meaningful topics depending on their semantic content. In this way data become relevant for the operators to make decisions. Two real cases used to test its applicability showed that different visualization techniques might be needed to support situation awareness. This ontology-based approach can be generalized for analyzing the information flow about other domains of application changing the underlying knowledge base.

## Background

Nowadays, technological advances have equipped most citizens with innovative and very powerful devices that can be used almost everywhere to communicate quicker and broader than ever. As stated by Westlund in 2013, *mobile devices are used for reporting live from both everyday life events and more significant events* (Westlund 2013). This phenomenon is also called *citizen journalism* and it concerns posting all kinds of information on social networks or blogs, including text messages, photos, videos or GPS locations. The volume of shared data grows significantly in case of critical events. For example, during the Sandy hurricane on November 2nd, 2012, the official Twitter account announced that *people sent more than 20 million tweets about the storm between Oct 27 and Nov 1* tracking the words *hurricane* and *sandy* as well as the hashtags #hurricane and #sandy. More recently, during the first hour after the Germanwings plane crash on March 24th, 2015, 60 thousands of messages containing the hashtag #Germanwings were posted on Twitter (Hodgson 2015).

The messages posted on social networks can represent a useful source of information for emergency operators in charge of making decisions about activities to perform and resources to use, as stated in Lindsay (2010). Moreover, they can be considered as a common communication channel established between emergency operators and citizens for sending and receiving details about the situation. This channel can be also used for encouraging citizens to play a more active role depending on their profile and previous experiences, as modeled by Díaz et al. (2013).

Considering the great volume of information generated by citizens, it becomes more and more necessary to provide emergency operation centers with specific tools for analyzing and tracking these data. Several contributions apply visual analytics to the collected messages. They mainly focus on what people is saying in social networks, extracting opinions together with other useful details, like time, geolocalization and word frequency, but in a domain like emergency management this is not enough. Whenever a crisis happens, thousands of data are generated by citizens, but many of them are irrelevant, inaccurate or even false. One of the main challenges that emergency management agencies and corps face to integrate social networks in their practice is the lack of resources to extract meaningful knowledge from such a heterogeneous mass of data (Díaz et al. 2014). Therefore, a tool is needed to analyze the information flow from social networks and to extract the most relevant knowledge respect to a specific scope, as for example the evolution of a critical event.

Emergency operators are interested in extracting just useful content and identifying most critical information (Palen and Liu 2007). But, how to filter data from social networks to extract most meaningful topics for emergency operators’ purposes? To answer this question, in this paper we propose a semantic approach for monitoring social network activities during a crisis. Our approach consists of four different steps: (1) data collection, (2) syntactic analysis, (3) semantic model, (4) categorization. The first one collects messages from social networks about a specific topic. The second one processes these messages from a syntactic point of view identifying frequencies and speech functions. The third and the fourth ones extract and categorize most relevant terms using data mining techniques based on a semantic model. The applied semantic model is an existing ontology built for representing the correlation among four knowledge domains, including emergency, evacuation, technology and accessibility (Malizia et al. 2010). In this way, data are filtered out automatically and covered by domain specific knowledge so they can be presented in a more meaningful way to the operator. For instance, all the posts related with evacuation procedures or resources requests can be grouped under these categories so the exploration is made easier.

To test the applicability of this approach, we present two real case studies: the Nepal earthquake of April 25th, 2015 and the hurricane Sandy of October 27th, 2012. While during the Nepal earthquake we have collected 822 tweets identifying 116 relevant terms, the hurricane Sandy data set consists of almost 500,000 tweets and 5500 relevant terms. Applying our approach to these real cases, we show how different kinds of visualizations are required depending on the collected data. In particular, we notice that the visualization technique rely upon not only on the volume of data but also on the existing interrelationships among them in order to support some level of situation awareness in decisions makers. Situation awareness has been defined as *the perception of the elements in the environment within a volume of time and space, the comprehension of their meaning and the projection of their status in the near future* (Endsley 2003). The goal will be then to provide a visualization that might help decision makers in understanding better what’s happening in a specific situation from the analysis of the information that citizens are generating in the social networks. Our approach to improve such understanding is to rely upon semantics to categorize tweets and, therefore, make them more meaningful avoiding trivial information. In this way, we propose an automatic tool to manage data flowing from social networks that operators can easily interact with and draw conclusions about what is happening.

## Social networks usage in emergency and crisis situation

Information posted on social networks becomes an important source for knowing most discussed topics among a community of people. In this way, social interactions have been translated from the physical communities to the virtual ones. Thanks to the technological advances that make it possible to connect different kinds of devices all over the world through the Internet, these virtual interactions are growing for both personal and business purposes. They work as a new communication channel for sharing a great quantity of data, including photos, videos or text messages. Another interesting characteristic of social networks is that connected users can be reached in just few moments even if they are geographically distributed in different places.

Kleinberg (2008) defines this information spread as an *epidemic wave* that could represent a powerful influence for changing the opinions and the behaviors of people. This phenomenon can be observed for example in case of political elections, where social networks are used as part of candidates’ campaign. Another interesting application is the great volume of information shared when a critical event occurs, like earthquakes, tornadoes or terrorist attacks. Posted messages generally are about feelings and situations that people are experiencing. This sharing activity has been analyzed by Alexander (2013) with the identification of seven different usages of social networks in emergencies: listening to public debate, monitoring situations, extending emergency response and management, crowd-sourcing and collaborative development, creating social cohesion, furthering causes (including charitable donation) and enhancing research.

How social networks have been employed during crisis is the aim of an extensively review of Reuter et al. (2012). The first analyzed use case is the terrorist attacks of 9/11 (September 11th, 2001), when citizens created wikis looking for useful information about missing people. The same behavior has been observed in other situations with the usage of different social networks, like Twitter, Facebook, YouTube or Flickr, as direct communication channel among citizens. Authors conclude the review proposing a model of this bidirectional channel between a receiver and a sender, where both of them can be official emergency organizations or common citizens.

Another interesting report about the usage of social media by governmental agencies and operation centers (Lindsay 2010) concludes that social media open new opportunities within the emergency management by establishing a direct communication channel between operators, victims and witnesses in a simple way. From the citizens’ perspective, they can actively participate during the response activities whilst decision makers can use collected information to know more detailed data about human and physical damages (Díaz et al. 2014). Nevertheless, there are some limitations to take into account like for example the trustability of posted information and the consequent need of specific policies for guaranteeing the privacy (Hiltz and Kushma 2014). Indeed, a study performed with professional EM workers in the area of British Columbia (Canada) and in Spain showed that the main issues that might deter official agencies to use social media is the lack of resources to keep up with such a huge quantity of data, and to be able to make sense of it (Díaz et al. 2014). Next section reviews some visual analytics tool designed to cope with this problem.

## Visual analytics tools for social networks: a comparative study

Messages published on social networks represent a useful source of information for different purposes, including news spread or emergency management, as already explained in the previous section. Depending on the topics of interest, the volume of this source grows rapidly as new content is published and keeping track of it has become more and more complex.

The most common solution to this problem has been the development of visual analytics tools for monitoring and exploring the data collection. As defined in Thomas and Cook (2005), these tools are used *to synthesize information and derive insight from massive, dynamic, ambiguous, and often conflicting data*. To do so, they combine information visualization techniques with data mining methods. The result is an inclusive experience for the users that can explore collected data and eventually access to more details.

In order to understand advantages and disadvantages of this approach, we have selected twelve tools as the most relevant contributions proposed in literature during the last 5 years. As shown in Table 1, for each one we have studied three aspects: (1) main features, (2) proposed visualizations and (3) data mining techniques. In particular, our analysis focuses on how this last aspect is applied. Indeed, data mining supports the identification of relevant content respect to a specific data set. In case of emergency management, it plays a crucial role for avoiding misunderstanding and time wasting for operators in charge of making quickly critical decisions.

The Vox Civitas (Diakopoulos et al. 2010) is a visual analytics tools for extracting the most common opinions over social networks and relating them with broadcasting news. Following a more journalistic perspective, it correlates a video source with tweets, specifying for each moment the number of posted messages and the most shared sentiment. Using the U.S. State of the Union of 2010 as case study, Vox Civitas allows to identify the most commented minutes of Obama’s discourse as well as the sentiments of shared posts on Twitter. Collected messages are categorized measuring the term-similarity and extracting their relevance in relation to a specific minute of the video. The sentiment analysis is based on a two-steps process. First of all, some common words for positive and negative opinions are recognized. After that, a learning model is applied based on a trained set of 1900 messages manually tagged. Nevertheless, Vox Civitas doesn’t cover the selection of tweets related to a specific event.

Another tool, called Visual Backchannel (Dork et al. 2010), focuses mainly on proposing different visualization techniques: a streamgraph of the evolution of a topic over time; a helical graph of the participation of most active users in posting messages; a list of tweets; a cloud grouping all the shared images. From a semantic point of view, the performed text analysis is based on reducing each term in a tweet to its root form (e.g. *animals* to *animal* and *eating* to *eat*) and computing its frequency for identifying the most relevant ones.

Hao et al. (2011) have introduced three innovative sentiment analysis techniques. The first one focuses on understanding users’ opinions. The second one extracts the most relevant posts considering locations, sentiments and other characteristics, like retweets and followers. The last one shows the geographical distribution of collected data over a map. These techniques aim at identifying useful patterns and influences that tweets could show on the market. Also in this case, authors apply a natural language processing method for analyzing semantically the messages for the opinion mining.

SensePlace2 (MacEachren et al. 2011) offers an intuitive interface where users can query a topic and obtain a list of tweets. They can also specify a time range and select the most relevant messages. The relevance measure is based on the term frequency and it is also used for clustering the geographical distribution of the tweets over a heat map. The users can also interact with the map and choose a place for visualizing tweets published there.

Cao et al. have proposed Whisper Cao et al. (2012) for exploring how information and opinions about a given event or topic spreads. In particular, they present an innovative visualization technique based on a sunflower metaphor. The bloom of the sunflower represent tweets of interest, while the seeds that are dispersed by the wind or animals are the diffusion paths. The seeds are then gathered and eventually dispersed again (i.e. retweeted or favorited) by clusters of users called communities. In this way, Whisper allows to relate among each other the activities of communities, the geographical distribution of posts, and users’ opinions.

In 2012, Yin et al. have analyzed the effect of the volume of data generated on Twitter over the emergency management activities with the development of an intelligent system for capturing automatically any burst of posts. Collected tweets are then manually tagged to select the ones with information about a specific event. Finally, two visualizations are proposed: a map with a geographical clustering of the messages, and a tag cloud with a time slider where users can explore most relevant terms at a given time range.

TweetXplorer (Morstatter et al. 2013) offers three different visualizations for answering to four basic questions in information gathering: who, what, where, and when. The first adopted technique is the retweet network as a representation of most relevant users and tweets. The second one is a heat map, where both size and color of each circle change depending on the number of posts from a specific place. Finally, a tag cloud is displayed with the most frequent terms used by users for describing the event.

Liu et al. (2014) have presented TopicPanorama, where messages posted on different social platforms about the same topic are merged in a density-based graph. The graph combines a pie chart for each topic and its relevance over the considered sources and a hierarchical structure for mapping the topics among each other. Exploring the resulting graph, the users can easily identify the most discussed topics over social media. Moreover, TopicPanorama allows to modify the mapping and the topics depending on the specific needs of the analyst that is using it.

A system called OpinionFlow (Wu et al. 2014) has been developed in 2014 for extracting public opinions. Through a strip graph and a timeline, authors offer a platform where analysts can select a topic of interest and visualize the opinion flow among Twitter users. Each strip is represented as a diffusion model for strong and weak as well as positive and negative opinions. Another feature of the system is to compare opinion flows related to different topics and identify diffusion patterns to be used for application like marketing or public debates.

Opinion mining is also the scope of Matisse, an intelligent system developed by Steed et al. (2015). Apart from opinions, it provides also other information, as term frequency, time range and geographical position. All these data are combined into three linked visualizations: a timeline, a streamgraph and a heat map. Users can navigate and explore them as well as use some filters for selecting messages posted in a specific time range and place.

The prototype by Zimmerman and Vatrapu (2015) consists of six different dashboards where from different social media are combined. It is called Social Newsroom and it into six different dashboards. Three of them have been designed for presenting offline statistics about most relevant topics, sharing activities, *likes* from other users, and most active contributors in the networks. The other three dashboards monitor the same information real-time, giving a more detailed view of the message evolution.

The last visual analytics tool included in our analysis is ScatterBlogs (Thom et al. 2015). From a dataset of tweets collected about an event, it proposes different analysis. First of all, messages are shown in a map and users can apply a content lens for generating a tag cloud of most frequent terms in a specific place. Secondly, messages are clustered based on the similarity of their content and geographical position. In this way, it is possible to easily recognize the most critical events that generate a great volume of data. Finally, a sentiment analysis is performed and shown in a timeline. Provided visualizations are linked in order to allow an effective exploration of represented information.

**Table 1:** Visual analytics tools for social networks

Tool	Functionality	Visualization	Semantic
Vox Civitas Diakopoulos et al. (2010)	Filtering messages Relevant terms Unique messages Sentiment analysis Keyword extraction	Video Volume graph Timeline Bar graph	Similarity Learning model Stemming
VisualBackchannel Dork et al. (2010)	Text analysis Topic identification	Streamgraph Helical graph Image cloud	Stemming
Hao et al. (2011)	Sentiment analysis Stream analysis Visual analytics	Timeline Map	NLP techniques
Senseplace2 MacEachren et al. (2011)	Filtering tweets Relevant tweets History view	Timeline Map Heatmap	None
Whisper (Cao et al. 2012)	Monitoring User exploration Filtering	Timeline Sunflower	None
Yin et al. (2012)	Data capture Burst detection Text classification Online clustering Geotagging	Date-time slider Tag/topic cloud Map	Manual
TweetXplorer Morstatter et al. (2013)	Grouping keywords Timeline Relevant users/tweets Geotagging User patterns	Retweet network Heat map Tag cloud	None
TopicPanorama Liu et al. (2014)	Topic analysis Matching social media	Radial tree Density graph	Ranking
OpinionFlow Wu et al. (2014)	Opinion mining Diffusion model Relevant users/topics	Strip graph Timeline	Opinion mining
Matisse Steed et al. (2015)	Sentiment analysis Geotagging	Timeline Scatterplot Heatmap	Learning model
Social Newsroom Zimmerman and Vatrapu (2015)	Cross-platform Story success Relevant users/topics	Statistical graph	None
ScatterBlogs Thom et al. (2015)	Filtering tweets Event discovery Topic model Tag clusters	Content lens Tag cloud Timeline	NLP techniques

Analyzing the features provided by each one of these tools, we have recognized three main purpose-oriented categories: *Opinion mining*, *Relevance*, and *Monitoring*. The scope of the *Opinion mining* tools is to identify positive and negative sentiments of people about most discussed topics in social networks. This information is useful for that applications where end users’ opinions could influence decisions to make or activities to perform. The *Relevance* class aims at looking for that topics and terms that are significant for understanding what people is talking about. For example, if we are collecting information about a natural disaster, terms like *quake* or *collapse* are more relevant respect to support expressions like *prayer* or *donation*. Depending on the applied data mining techniques, results could be more or less precise. In the *Monitoring* class we include tools for exploring social networks content and keeping track of shared information and its evolution on time and geographical distribution. In this way, it is possible to obtain some interesting analysis about users’ activities on social networks, like for example from where users are mostly posting.

From a semantic point of view, we have detailed this categorization considering the implemented techniques for data mining, as shown in Table 2: (1) *None*, where any analysis is performed over collected messages; (2) *Manual*, where just a sample of the entire collection of messages is analyzed manually by domain experts; (3) *Syntactic*, where syntactic information as the term frequency or its part of speech is extracted; and (4) *Natural Language Processing* or NLP, where language models are applied for obtaining sentiments and relevant terms from a text. The result is that six tools apply NLP methods, four of them do not consider any data mining technique, the contribution of Yin et al. involves some domain experts for analyzing manually collected messages from social networks, while VisualBackchannel implements some syntactic algorithm for filtering stop words using the part of speech.

In these paper we try to go a step forward and analyze data from the perspective of the emergency operator who has to make sense of the visualization as soon as possible to make informed decisions. For that to be possible, relevant terms are not necessarily those more frequently mentioned but those that are related to meaningful and decisive terms to understand the situation. As posited by Hoffman and Oliver-Smith (2002), disasters are multidimensional and each person describes them according to how they experience them. However, such description is not necessarily useful for the operator. To cope with this fact, we propose to analyze the information generated by citizens by filtering it out using a domain ontology that collects meaningful terms in the emergency domain. In this way, information can be organized according to relevant categories that make sense for the operator.

Considering the amount of published tweets, if a user wants to share her message with as many people as possible, she has to find an effective strategy like mentioning accounts with a great number of followers. In Twitter (2015), several best practices for using Twitter are collected. Among them, it is presented the case of a user looking for her sister after the Japanese earthquake of 2011. In order to be more effective, she asked a NBC TV show for help and just in one day she reached her sister safe and sound. The emergency operators have the same need of this user to stand out just the most crucial and useful messages through the generated information flow.

**Table 2:** A comparison of considered visual analytics tools

Type	None	Manual	Syntactic	NLP
Opinion mining				Vox civitas Hao et al. OpinionFlow Matisse
Relevance	Senseplace2 TweetXplorer Social Newsroom		VisualBackchannel	TopicPanorama ScatterBlogs
Monitoring	Whisper	Yin et al.		

## Using semantic modeling techniques for Twitter

Our approach consists of analyzing semantically messages collected from Twitter related to a specific event so they can be made more meaningful by a categorization based on terms from the cognitive framework of decision makers. In this way, we propose a model for defining a knowledge ecosystem (Thomson et al. 2007) where different kinds of data are interpreted and categorized depending on their meanings and relevance. This is the result of a four step mechanism based on an existent ontology and taxonomies, already presented in Onorati and Diaz (2015).

**Fig. 1:**
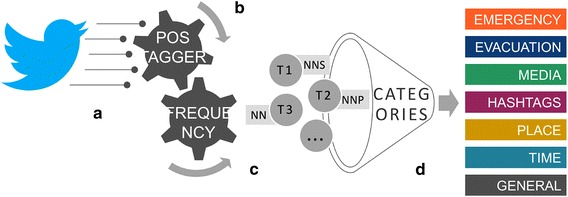
Semantic modeling technique for categorizing information generated from twitter: **a** search query; **b** POS tagger; **c** frequency filter; **d** semantic categorization

The first step, (a) in Fig. 1, queries Twitter for one or more keywords (e.g. Nepal, earthquake, hurricane, Sandy) through the Search API available as part of the Twitters v1.1 Rest API. Collected tweets are then analyzed syntactically in the second step, (b) in Fig. 1. The syntactic analysis consists in applying an algorithm called POS (Part Of Speech) for extracting proper and common nouns in their root form (e.g. earthquake, emergency and NYC). Considering that each tweet has a limit of 140 characters, people use few words for writing their feelings and opinions. For this reason, we posit that contained nouns could carry the most meaningful information respect to the other parts of speech, like verbs or adjectives.

The result of the second step is a large list of nouns that needs to be filtered for identifying the most relevant ones for the query search. For achieving this objective, in the third step, (c) in Fig. 1, the nouns are ordered depending on their frequency in the collection of tweets while in the fourth step, (d) in Fig. 1, they are semantically related to seven fixed categories. These categories have been chosen as relevant issues to take into account within the domain application of this work: *emergency*, *evacuation*, *media*, *place*, *time*, *hashtags* and *general*. In particular, they came from the six journalistic questions: who did that (general and emergency), what happened (hashtags, emergency and evacuation), where did it take place (place), when did it take place (time), why did that happen (emergency) and how did it happen (emergency).

To relate the nouns extracted from Twitter with the seven categories, we use different techniques. In *hashtags* we collect all the labels composed by a pound sign (#) and a word (i.e. #hurricane) and in *general* all the uncategorized nouns. For the remaining five categories, we consider the highest semantic similarity. To do so, each category is represented by a set of meaningful and relevant concepts that then are semantically compared with the list of nouns.

For the conceptualization, we use different semantic models depending on the knowledge related to the category. The first one is an existing ontology called SEMA4A (Simple EMergency Alerts 4[for] All) that has been developed for correlating users needs, technologies and relevant information about emergencies (Malizia et al. 2010). The SEMA4A ontology contains several concepts organized in a vertical hierarchy with four main classes: emergency, evacuation, accessibility and communication. Each concept has a set of meanings and ad-hoc relations with other concepts. For example, evacuation has an *include* relation with map and typhoon has a *kind of* relation with emergency. Within our scope, we use the three classes emergency, evacuation, and communication of SEMA4A to conceptualize respectively *emergency*, *evacuation* and *media*. In particular, *media* has concepts related to the kind of content, like photos, videos or audios, and the used communication channel, like radio, television or Internet. The *time* and *place* categories are instead represented by two different taxonomies: the first one has about 150 time expressions like day, after and later, while the second is an open source list of all countries and cities in the world.

The semantic similarity is a binary measure with two results, *similar* or *not similar*. In this work, we are going to use it for relating a term with one of the considered categories. To do so, we apply a two step technique using the semantic models (i.e. an ontology and two taxonomies) previously described as knowledge representations for the categories. Taking as an example a word *w* coming from Twitter, during the first step we check whether *w* matches with one of the words included in the semantic models. If yes, *w* is going to be assigned to the represented category. If not, we execute the second step where first of all we extract from a lexical database called WordNet (Miller et al. 1990) a set of synonyms $s_{1}$, ..., $s_{n}$ with the same meaning of *w*. At this point, for each $s_{i}$ we check if it has a match within one of the semantic models. If yes, for the synonym relation we can infer that *w* is similar to the represented category. if also this last step fails, we conclude that the word *w* cannot be related with any category and consequently it is going to be grouped into the *general* one.

As an example of the semantic similarity measure, let consider the word *nyc*. This is an acronym standing for New York City. Following the two step technique, we look for any match with the terms included in the semantic models. Failing this step, we extract the synonyms from WordNet and among them we find the acronym *US*. Looking for it in the semantic models, we find a match with the taxonomy representing the *place* category. Consequently, we associate *nyc* with it.

At the end of the fourth step of the proposed mechanism, we obtain a categorization of the most relevant words collected from Twitter. The main contribution of this approach is the combination of both a syntactic technique based on the frequency and the part of speech and a semantic method with several knowledge models for representing the relevance of the information flow.

## Case studies

The proposed semantic modeling technique has been developed as an off-line tool for analyzing tweets once they have been collected. Considering that emergency situations are generally unforeseeable event, in this way we have the possibility to test whether and how the approach could be applied in real cases. We have chosen two case studies that vary considerably in the number of information generated and they help also to illustrate potential visualization techniques that can be used to support data exploration. The visualization of the outcomes of this process plays a fundamental role in order to facilitate the interpretation by the operators. It will be the front-end application that emergency operators will interact with for searching, navigating and exploring information generated from Twitter in order to understand the situation and make the appropriate decisions.

One of the aspects to take into account to choose a specific visualization technique is the volume of data to be presented. Depending on how many concepts have been extracted and categorized, users could find more effective a technique instead of another. Here we are going to present two different visualizations: a Hierarchical Edge Bundle and a Bubble Chart. Other types of visualizations might be required in other scenarios, but the goal of this paper is not to describe all possible visualizations but to test whether semantic mechanisms help to organize information in a meaningful way.

The Hierarchical Edge Bundle focuses on the relation among topics as they have been shared by people. Related topics can help in clarifying what people refers to with their messages and contextualizing the information. Considering the specific characteristics of this technique, it is suitable to represent few terms and consequently a reduced number of tweets. We tried it in a previous contribution, where we studied the Nepal Earthquake event with a collection of 822 tweets, dated between the 25 and the 28 of April, 2015 (Onorati and Diaz 2015). Applying the semantic approach here presented, from an initial set of 1262 words the final categorization contained 123 terms organized as follows: 28 in emergency, 10 in evacuation, 1 in media, 5 in place, 17 in time, 9 in hashtags and 53 in general.

**Fig. 2:**
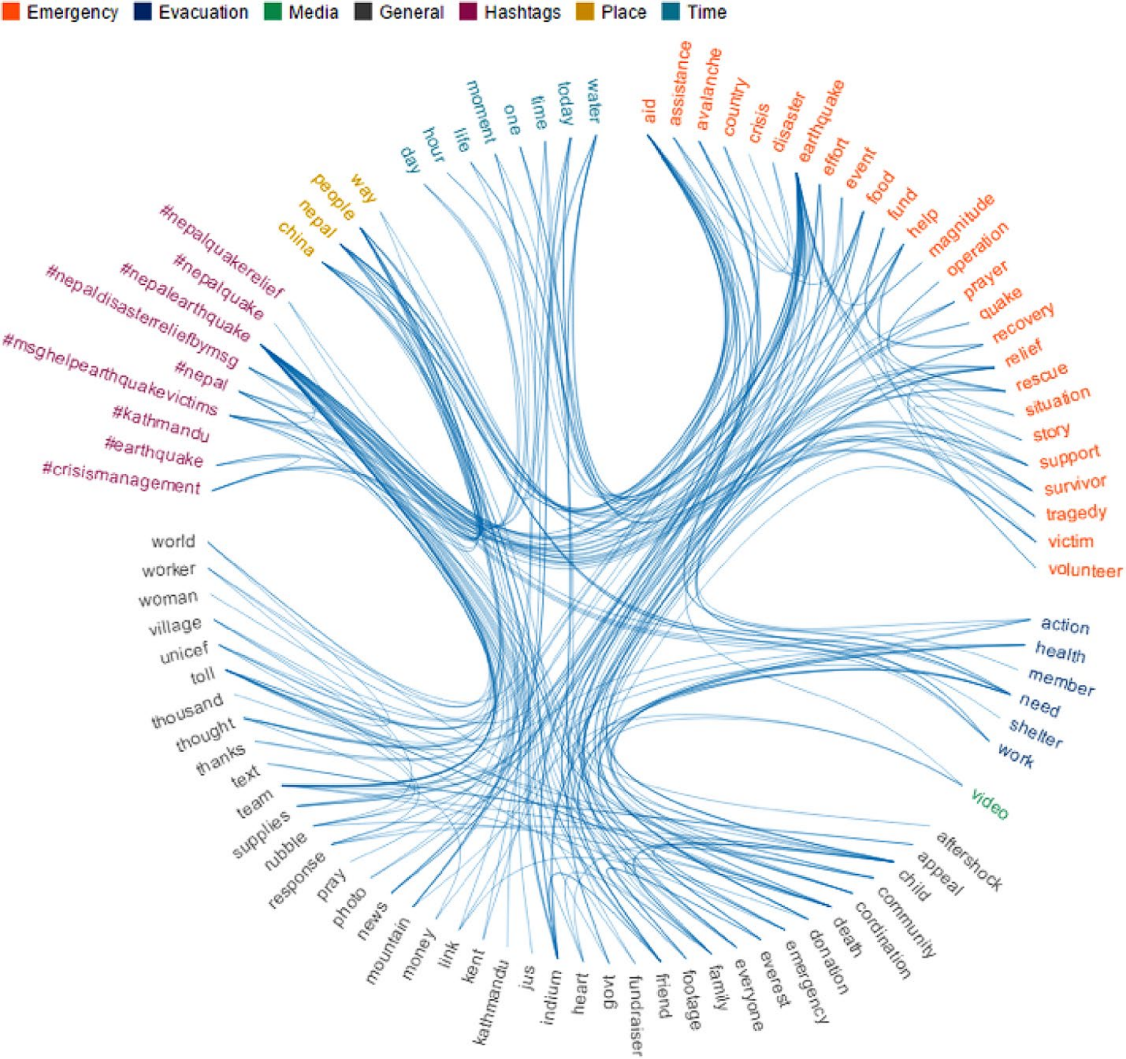
The hierarchical edge bundle for the Nepal Earthquake case study (Onorati and Diaz 2015)

In the Hierarchical Edge Bundle, categories are coded with different colors, as shown in Fig. 2, and terms are grouped all around a circle and linked depending on their co-occurrences in the same tweet. This technique makes it possible to draw some conclusions about visualized data. (1) Which are the most discussed topics/categories as the ones that receive the highest number of arcs (e.g. earthquake, aid or the hashtag #nepalearthquake). (2) Which terms are mostly used together for stating an opinion or a feeling as connections among different terms (e.g. people with aid and need). (3) Which kind of information is commonly shared during this event as the classes with a higher number of terms (e.g. general and emergency). (4) How the information flows from a topic to another as the categories that have the greatest number of links between each other (e.g. hashtags and emergency). For example, we can conclude that (1) users mostly discussed about topics related to general (e.g. team, death and child) and emergency (e.g. aid, earthquake and relief) categories. Moreover, (2) the hashtag #neaplearthquake and the nouns aid, earthquake, people, Nepal and relief are mostly used together as (3) people mainly tweet about the earthquake and how to help affected people. Finally, (4) one of the most interesting flow of information goes from #neaplearthquake to donation through concepts like team, people, aid and need showing how people started several fundraiser initiatives.

The Hierarchical Edge Bundle is useful in case of a small collection of information, but what happens if we have a greater volume of data to manage? This is the case of the Hurricane Sandy. At the end of the crisis more than 20 million tweets were published. These tweets are the result of querying keywords *hurricane* and *Sandy*, as well as hashtags *#hurricane* and *#sandy* during the hurricane hitting New York bay on October 29, 2012. Due to such amount of information, this data set is one of the most studied in literature as an example of large volume of critical information shared in few days. In this work, we run the proposed mechanism with a subset consisting of almost 500,000 tweets. Considering that the maximum number of tweets per minute was 16,000 (Imran et al. 2015), we select the subset as the messages generated during thirty minutes trying to simulate a real time interaction with the information flow.

Applying the semantic modeling technique, after the first syntactic analysis we have obtained an initial list of 24,000 nouns reduced to 5600 using the frequency based filter. Successively, the result of the semantic analysis gives the following categorization: 265 terms in emergency (e.g. hurricane occurring 2753 times), 75 in evacuation (e.g. home occurring 95 times), 23 in media (e.g. internet occurring 15 times), 1079 in hashtags (e.g. #sandy occurring 1207 times), 159 in time (e.g. day occurring 131 times), 179 in place (e.g. nyc occurring 327 times), and 3804 in general (e.g. apocalypse occurring 191 times).

Considering the number of terms contained in each category and the great difference generated among the relevance of considered terms, the Hierarchical Edge Bundle loses its effectiveness in this case. For this reason, we bet for a different technique called Bubble Chart. In Data Visualization, a Bubble Chart represents three dimensions of the same data point: the x and y coordinates in the space and the size of the bubble. In our context, each bubble represents a term where the size is its relevance respect to the search query. At this point, nouns included in the chart are the result of the previous filtering and categorizing phases and we can consider all of them relevant for the specific application. For this reason, the size of the bubbles corresponds to the term frequency over the entire collection of tweets. Used color for the categories are the same of the previous visualization: orange for emergency, blue for evacuation, green for media, purple for hashtags, yellow for place, sky-blue for time and gray for general.

The kind of conclusions that it is possible to draw here is mainly related to the relevance of both individual terms are general topics. The relevance in this case is represented by the position of the bubbles respect to the circular space. In this way, it is possible to observe that the most relevant term is *hurricane* right in the center. Moreover, bubbles from the same category create concentric circles of the same color that help in identifying the most discussed topics reading them inside out. We can observe that people use to share information about the emergency itself, like storm or rain (i.e. orange bubbles are right in the center of the visualization) and the evacuation details, like school, plan and home (i.e. blue bubbles are next to the orange ones) using also general concepts, like friend, family and wish (i.e. grey bubbles are all around the orange and blues bubbles). Finally, with the same relevance there are also hashtags like #sandy and #hurricane, media like phone, place like nyc and time like tomorrow (i.e. purple, green, yellow and sky-blue bubbles are on the same circumference all around the grey ones).

**Fig. 3:**
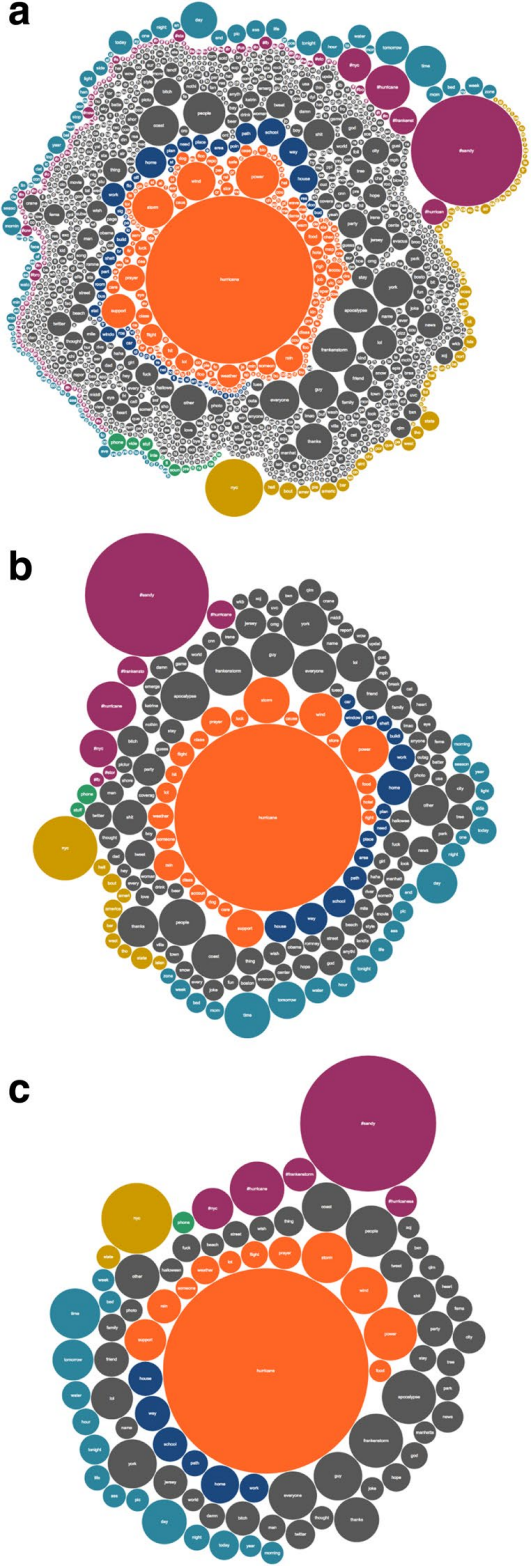
The bubble chart evolution for the hurricane Sandy case study: **a** minimum relevance of 1; **b** minimum relevance of 15; **c** minimum relevance of 30

To offer a more efficient navigation to the emergency operators, we also give the possibility to vary the number of bubbles shown in the chart. The users can select a different relevance range depending on the scope of their exploration. In Fig. 3, the Bubble Chart evolution is shown. For a more understandable interface, we decide to represent the relevance measure as a numeric value determined experimentally as included between 1 and 30: see a, b and c in Fig. 3.

Observing the Bubble Chart evolution, it is possible to note how the terms with a highest relevance are mostly contained in the specific categories of emergency, evacuation, hashtags and place. Changing the relevance measure, the most general terms disappear making the chart more readable. Moreover, the users can click on a bubble and list in a separate section the tweets with that term. In the future, we are studying the possibility to select more than one bubble and show tweets where the terms co-occur.

The application of proposed approach to the Nepal Earthquake and the Hurricane Sandy shows how emergency operators can take advantage of collected information through ad-hoc visualizations. While people mostly share general opinions and feelings about the emergency, operators can easily choose which data they want to explore further or recognize the most discussed topics, avoiding wasting their time with irrelevant facts. The situation awareness is in this way enriched by a knowledge model of semantically relevant data. In this way, we aim at improving the cognitive framework of decision makers with a better perception of the context and different levels of details depending on their needs for making decisions.

Another interesting advantage is about the usage of hashtags: these special words represent a useful guide for identifying relevant information flow. In particular, it is possible to relate them with other data, like time and geographical position, to understand how topics discussed by people evolve during the crisis. Emergency operators can use these details to monitor people’s reaction and execute the corresponding emergency protocols. This is what happens for the Hurricane Sandy during the final hours of October 29, when people started to use the hashtag #staysafe for sharing information about evacuation and safety procedures.

## Conclusions and future works

The citizen participation during critical events, like hurricanes or earthquakes, is an important aspect to take into account for improving the emergency response phase. In particular, the flow of messages generated by social networks represents an important source where it is possible to find interesting information about the emergency evolution or the rescue actions. The problem stands in the volume of shares, likes and posts published every moment in Internet: How can emergency operators make sense of them without losing time? The answer is an intelligent tool able to collect, analyze and extract relevant information for them.

In literature, several visual analytics tools have been developed offering different kinds of data analysis and visualization techniques. From our survey, we have found out a lack of a semantic perspective that could take into account the emergency operators’ point of view showing them just data that they really could need for making informed decisions as soon as possible. For this reason, in this paper we try to go a step forward performing a semantic analysis of collected dataset based on specific knowledge domain. The result is a categorization where the most relevant terms are related to one of the following seven concepts: emergency, evacuation, media, hashtags, time, place, and general. In this way, emergency operators can choose which topic they are more interested in or find some interesting conclusions about how the information flows from a tweet to another.

The proposed semantic modeling techniques is based on the assumption that each category is meaningful for representing the emergency search query. For relating each term extracted from the tweets, we use three different semantic models: an ontology with concepts about emergency, evacuation and media that has been already validated in Onorati et al. (2014), and two taxonomies about time expressions and names of places. These models can be considered a quite complete representation of the knowledge contained in each category. Our approach aims at abstracting the individual terms to contribute with a more general knowledge base that users can consider as part of their baggage.

Relying upon these semantic models, an intelligent tool has been designed to be easily integrated in the emergency center avoiding any distractions for the operators or waste of resources and time. In this way, the proposed solution becomes an effective support for making decisions or discovering new information.

From a theoretical point of view, the performed semantic analysis is based on standard data mining techniques that make it easy to generalize or adapt for other domains of applications, like politics or marketing. In this last case, other researchers could apply our ontology-based methodology changing adequately the knowledge base to obtain meaningful topics according to predefined relevance criteria.

Another interesting outcome from this research concerns the visualization technique. As shown in the two case studies of Nepal Earthquake and Hurricane Sandy, the visualization technique has to be chosen depending on the volume of generated data and their semantic distribution in the categorization. The chosen techniques are an example of a possible interpretation of collected information. In the future, we are planning to try different visualizations with the same data-sets and compare them during a qualitative evaluation with emergency workers in operational centers.

The mechanism proposed in this work can be applied both off-line during the recovery phase to collect as much information as available about victims and damages, as shown with the case studies, and real-time. To be used real-time, the four step mechanism will be executed over a dynamic data-set each time a new tweet or a set of new tweets are published about an event. Consequently, the visualization can change giving the possibility to the emergency operators to work also on the evolution and the dynamism of the situation. The next step of this research work is to test our approach real-time during a large scale emergency situation involving also domain experts. In this way, we will be able to evaluate not only the usability of the tool but also its performance in term of running time.
